# Structural brain changes related to bilingualism: does immersion make a difference?

**DOI:** 10.3389/fpsyg.2014.01116

**Published:** 2014-10-02

**Authors:** Maria Stein, Carmen Winkler, Anelis Kaiser, Thomas Dierks

**Affiliations:** ^1^Department of Psychiatric Neurophysiology, University Hospital of Psychiatry and Psychotherapy, University of BernBern, Switzerland; ^2^Department of Clinical Psychology and Psychotherapy, Institute of Psychology, University of BernBern, Switzerland; ^3^Center for Cognition, Learning and Memory, University of BernBern, Switzerland; ^4^Department of Social Psychology, Institute of Psychology, University of BernBern, Switzerland

**Keywords:** bilingualism, naturalistic learning, immersion, second language, structural plasticity, VBM, DTI

## Abstract

Within the field of neuroscientific research on second language learning, considerable attention has been devoted to functional and recently also structural changes related to second language acquisition. The present literature review summarizes studies that investigated structural changes related to bilingualism. Furthermore, as recent evidence has suggested that native-like exposure to a second language (i.e., a naturalistic learning setting or immersion) considerably impacts second language learning, all findings are reflected with respect to the learning environment. Aggregating the existing evidence, we conclude that structural changes in left inferior frontal and inferior parietal regions have been observed in studies on cortical gray matter changes, while the anterior parts of the corpus callosum have been repeatedly found to reflect bilingualism in studies on white matter (WM) connectivity. Regarding the learning environment, no cortical alterations can be attributed specifically to naturalistic or classroom learning. With regard to WM changes, one might tentatively propose that changes in IFOF and SLF are possibly more prominently observed in studies investigating bilinguals with a naturalistic learning experience. However, future studies are needed to replicate and strengthen the existing evidence and to directly test the impact of naturalistic exposure on structural brain plasticity.

## INTRODUCTION

Experience-dependent changes in brain structure were first investigated in rodents placed in environmentally enriched versus very sparsely equipped standard cages. These early animal studies reported effects of environmental enrichment on brain weight (Rosenzweig et al., 1962) or cortical thickness (Rosenzweig et al., 1972) suggesting a structural adaptation process of the brain in response to experience. Since then, experience-dependent changes in human brain structure have been investigated in relation to various learning experiences, ranging from complex visuo-motor tasks like juggling to musical proficiency as well as to various aspects of language learning (see, e.g., May, 2011; Zatorre et al., 2012; Lovden et al., 2013).

A large proportion of the world’s population is estimated to be bi- or multilingual (Bialystok, 2010; Grosjean and Li, 2013) and the importance of the ability to communicate in more than one language is even increasing in a globalized world. A growing body of literature has thus investigated this fascinating human ability and its neural underpinnings on a functional as well as on a structural level. Within this body of literature, studies targeting structural correlates of only one precisely defined language component (e.g., speech sound perception or grammatical skills) allow to postulate a direct relation between this very language domain and local brain structure (Golestani et al., 2007; Pliatsikas et al., 2014a). Other studies use a more global approach and relate bilingualism or a measure of global second language proficiency to brain structure. More specifically, in the first case, these studies simply compare brain structure of bilinguals to that of monolinguals. Potential differences are then related to bilingualism or general second language proficiency. In the second case, these studies typically assess second language proficiency either by a variety of language tests or by overall scores such as school grades and relate these measures to brain structure. In the context of the present research topic on naturalistic exposure, this Mini-Review will summarize these studies on global L2-learning with a special focus on the question whether certain change patterns can be related to the environment in which the second language has been learned (naturalistic learning through immersion vs. classroom setting). Naturalistic language learning through immersion is characterized by high levels of L2-exposure and implicit learning; it is thus similar to L1 acquisition. In contrast, traditional L2 classroom instruction is mainly based on formalized training exercises and explicit instruction (e.g., Dahl and Vulchanova, 2014). Due to these differences, it is thus well conceivable that the structural change patterns evoked by these two learning types differ.

## SELECTION PROCESS FOR INCLUSION OF STUDIES IN THIS MINI-REVIEW

A systematic literature search was conducted in the databases PubMed/MEDLINE and Google scholar in April 2014. After having read through the resulting literature, appropriate studies were selected according to the following criteria: (1) published in a peer reviewed journal, (2) brain regions specifically associated with overall L2-acquisition, (3) original research articles, and (4) concentration exclusively on global language proficiency (vs. a single language domain). As bilinguals per definition have higher L2 proficiency than monolinguals, group comparisons between mono- and bilinguals were also considered as meeting this last criterion. Studies on aging effects were not considered in this Mini review. While 27 studies met criteria 1–3, a subgroup of eleven concentrated on global language proficiency. One of these, a post-mortem single case study (Amunts et al., 2004), differed significantly in methodology and was therefore excluded. Ultimately 10 research papers met all four criteria and were included in this Mini-Review (see Li et al., 2014 for a more extensive review). All studies investigated structural brain changes related to overall second language proficiency. In the following, the studies will be grouped according to their focus on either gray matter (GM; **Table 1**) or white matter (WM) changes (**Table 2**) and will be discussed with respect to main findings and inferences that might be drawn regarding immersion into a second language.

**Table 1:** Overview of studies investigating gray matter changes related to global second language proficiency.

Author	Sample	Learning environment	Method	Analyses	Main results
Mechelli et al. (2004)	(A) 25 early and 33 late bilinguals; 25 monolingual controls	Early bilinguals: naturalistic setting Late bilinguals: mixed (class-room setting for some, naturalistic learning for others)	VBM (GMD)	Cross-sectional group comparisons (bilingual vs. monolinguals, whole-brain approach)	l-IPC: bilinguals > monolinguals
	(B) 22 bilinguals	Unknown	VBM (GMD)	Correlation of VBM-changes with proficiency and AOA in bilinguals (whole-brain approach)	l-IPC: positive correlation with L2-proficiency l-IPC: negative correlation with L2-AOA
Osterhout et al. (2008)	Four students learning Spanish in a university course	Non-naturalistic, classroom setting	VBM (GMD)	Pre–post-comparison in l-IPC (ROI-approach)	l-IPC: increases from pre to post
Stein et al. (2012)	10 exchange students learning German	Naturalistic setting	VBM (GMD)	Correlation of VBM-changes with proficiency-changes (whole-brain approach)	GMD-increase in l-IFG correlates with individual increase in proficiency
Martensson et al. (2012)	14 conscripts in the interpreter academy; 17 monolingual controls	Non-naturalistic setting	Cortical thickness, subcortical gray matter volume	Group comparison (interpreters vs. controls, whole brain approach) of cortical thickness and subcortical gray matter changes (pre–post) Correlation of brain changes with proficiency changes (ROI-approach based on group comparison)	Interpreter (vs. controls) showed higher increase in cortical thickness (pre–post) in l-MFG, l-IFG, l-STG and in bilateral hippocampal volume Proficiency correlated with increase in r hippocampus and l-STG
Ressel et al. (2012)	22 Catalan-Spanish bilinguals; 22 Spanish monolinguals	Naturalistic setting	VBM; volumetric measurement of HG	Group comparison (bilinguals vs. monolinguals, whole-brain brain approach) of VBM values. Group comparison of manually segmented HG volumes	No VBM differences at corrected threshold. Bilinguals had higher HG volumes than monolinguals
Klein et al. (2014)	22 simultaneous bilinguals; 22 early sequential bilinguals; 22 late sequential bilinguals; 22 monolinguals	Naturalistic setting	Cortical thickness	Cross-sectional group comparisons (bilingual vs. monolinguals)	Cortical thickness in l-IFG late bilingual > monolingual and early bilingual > monolingual Cortical thickness in r-IFG in monolingual > late bilingual, monolingual > early bilingual Simultaneous bilingual > late bilingual and early bilingual > late bilingual

*VBM, voxel-based morphometry; GMD, gray matter density; l, left; r, right; IPC, inferior parietal cortex; IFG, inferior frontal gyrus; MFG, middle frontal gyrus; STG, superior temporal gyrus; HG, Heschl’s gyrus; ROI, region of interest.*

**Table 2:** Overview of studies investigating white matter changes related to global second language proficiency.

Author	Sample	Learning environment	Analyses	Method	Main results
Coggins et al. (2004)	12 Bilinguals (seven early bilinguals; five late bilinguals); seven monolinguals	Classroom setting	Compare CC morphology (regional to total area ratio) between groups	Analyses of size (regional to total area ratio) of five CC subregions as defined on the midsagittal plane of an MRI image	AMB ratio to total CC: larger in bilinguals than in monolinguals
Mohades et al. (2012)	Children (8–11 years): 15 simultaneous bilinguals; 15 sequential bilinguals; 15 monolinguals	Simultaneous bilinguals: naturalistic Sequential bilinguals: classroom-setting	Group comparison of four preselected white matter tracts (AF/SLF, IFOF, AC-OL, AMB)	DTI	Left IFOF: FA in simultaneous bilinguals > sequential bilinguals > monolinguals AC-OL: FA in simultaneous bilinguals < sequential bilinguals < monolinguals
Schlegel et al. (2012)	11 English speaking students learning Chinese; 16 monolingual controls	Classroom setting	Longitudinal study comparing DTI changes in second language learners to those of mono-linguals	DTI (FA and RD)	Progressive FA increase in second language learners > controls FA increase in tracts connecting bilateral IFG, FMG, FPG, CN, left STG, PP, right PT FA increase related to second language proficiency
García-Pentón et al. (2014)	13 Spanish monolinguals; 13 Spanish-Basque bilinguals	Naturalistic	Group comparison of connectivity differences (whole brain approach)	DTI-based connectivity analysis, network based statistics, graph analysis	Two networks show higher connectivity and more graph-efficient information flow: (a) left-sided network comprising SMG, STG, IFG, MSFG, INS. (b) network comprising right SFG, left SPG, ANG, STP, SOG

*CC, corpus callosum; IFOF, inferior occipitofrontal fasciculus; AF, arcuate fasciculus; SLF, superior longitudinal fasciculus; AC-OL, connection anterior corpus callosum and orbital lobes; AMB, anterior-midbody of corpus callosum; UNF, uncinated fasciculus; FMG, frontomarginal gyrus; FPG, frontopolar gyrus; CN, caudate nucleus; PP, planum polare; PT, planum temporale; SMG, supramarginal gyrus; STG, superior temporal gyrus; IFG, inferior frontal gyrus; (M)SFG, (medial) superior frontal gyrus; INS, insula; SPG, superior parietal gyrus; ANG, angular gyrus; STP, superior temporal gyrus; SOG, superior occipital gyrus; DTI, diffusion tensor imaging; FA, fractional anisotropy; RD, radial diffusivity.*

## GRAY MATTER CHANGES RELATED TO SECOND LANGUAGE LEARNING

A seminal study on structural changes related to L2-acquisition compared early (age of acquisition (AoA) < 5 years) and late bilinguals (AoA: 10–15 years) to English monolinguals (Mechelli et al., 2004). Gray matter density (GMD) was higher in the left inferior parietal cortex (l-IPC) in bilinguals compared to monolinguals. This increase was even more pronounced in early relative to late bilinguals. In a second sample of Italian-English bilinguals (AoA: 2–34 years) the increase in L-IPC correlated positively with the degree of L2-proficiency and negatively with AoA. Mechelli et al. (2004) thus presented first strong evidence for structural changes related to bilingualism. However, with respect to the cross-sectional design of the study, it remained unclear whether the observed changes were directly induced by the experience of learning another language. The first study approaching this question in a longitudinal design (Osterhout et al., 2008) measured MRI in four University students enrolled in a 9 week intensive (3.5 h/day) Spanish course at two points in time (at the beginning and the end of the course). Because of the small sample size, the authors conducted a region-of-interest analysis in l-IPC and reported increasing GMD over the course of L2-acquisition, suggesting that structural changes in l-IPC are experience-dependent.

In a study with very high immersion to a L2-environment, native English-speaking exchange students learning German in Switzerland participated in language proficiency tests and MRI-measurements once at the beginning of their stay and a second time about 5 months later (Stein et al., 2012). The individual amount of learning (i.e., the L2-test score difference between first and second measurement) correlated with the increase in GMD in the left inferior frontal gyrus (l-IFG) as well as in the left anterior temporal lobe (l-ATL). While additional analyses exploring the effects of maturation and general environmental enrichment could not rule out the possibility that the l-ATL-cluster is due to these effects, the l-IFG-cluster seemed to be specifically linked to increasing L2-proficiency. The l-IFG changes thus reflected the individual amount of L2- learning (regardless of absolute proficiency). Martensson et al. (2012) investigated L2-acquisition through intense classroom-instruction and examined conscripts in the interpreter academy of the Swedish military, where a new language is learned to fluency within 10 months. These interpreters and monolingual controls participated in MRI-measurement immediately before the interpreter academy started and 3 months later. The grade on the mid-year exam (taken a few weeks after the second MRI-measurement) served as an indicator for language proficiency. Compared to controls, interpreters displayed larger pre-to-post-increases in cortical thickness in left middle frontal gyrus (l-MFG), l-IFG, left superior temporal gyrus (l-STG) as well as larger increases in hippocampi volumes. Furthermore, changes in right hippocampus and l-STG cortical thickness correlated with L2-proficiency level. Klein et al. (2014) compared cortical thickness of simultaneous bilinguals (AoA 0–3 years), early sequential bilinguals (AoA 4–7 years), and late sequential bilinguals (AoA 8–13 years) to monolingual controls. Interestingly, they observed differences in cortical thickness in l-IFG (higher thickness in bilinguals) and r-IFG (lower thickness in bilinguals) when comparing early and late bilinguals to monolingual controls, while no brain region differed significantly between simultaneous bilinguals and monolingual controls. Comparing Spanisch-Catalan bilinguals to Spanish monolinguals, Ressel et al. (2012) found no significant differences in a whole-brain VBM-analysis, but observed larger bilateral Heschl’s gyrus in the bilinguals.

## WHITE MATTER CHANGES RELATED TO SECOND LANGUAGE LEARNING

Most studies on WM changes used diffusion tensor imaging (DTI) to measure amount and directionality of water diffusion. When this diffusion is restricted in one direction more than in another (e.g., by an axonal cell membrane), water diffusion becomes anisotopic. Fractional anisotropy (FA) indicates to which degree water diffusivity is unimpeded (low FA) or restricted (high FA). FA values are typically higher along axonal bundles and thus allow investigating connectivity in the human brain (Conturo et al., 1999; Le Bihan et al., 2001).

To observe how bilingualism impacts WM pathways, Mohades et al. (2012) recruited simultaneous bilingual children (L2-exposure since birth), sequential bilingual children (AoA > 3 years) and monolingual children. Mean FA was assessed in four selected tracts with relevance to language processing. Higher FA-values were found in the left inferior frontal-occipital fasciculus (l-IFOF) in simultaneous bilinguals compared to sequential bilinguals and monolinguals. Furthermore, in the bundle arising from anterior corpus callosum (CC) and projecting into the orbital lobe (AC-OL) simultaneous bilinguals displayed lower FA-values than monolinguals. While the l-IFOF-finding suggests faster transmission of semantic information in simultaneous bilinguals (Duffau et al., 2005; Mandonnet et al., 2007), the interpretation of the AC-OL finding remains unclear (Mohades et al., 2012). Coggins et al. (2004) analyzed the variability of CC and compared the relative size of CC-subregions in bilinguals compared to monolinguals. The authors observed larger relative anterior-midbody CC in bilinguals and interpret this in the light of greater processing demands of multiple languages which require stronger interhemispheric communication of cortical regions bridged by anterior-midbody CC.

A recent study by García-Pentón et al. (2014) investigated anatomical connectivity in early Spanish-Basque bilinguals and native Spanish monolingual controls using a DTI-based tractography technique and network-based statistics. Bilinguals displayed increased connectivity in two networks: One left hemispheric network connecting frontal, parietal and temporal regions, and one network involving left occipital, temporal and parietal regions as well as right superior frontal gyrus. Within these networks, a graph-analytic approach indicated that in addition to higher connectivity, there is also more efficient information flow.

While the studies above all represent group comparisons investigating long term changes in WM, Schlegel et al. (2012) opted for a more dynamic approach to observe WM-changes in adults learning a new language. In this longitudinal study, monthly DTI scans were collected from English speaking students enrolled in a 9 month intensive Chinese course and from controls. Chinese-learners displayed a significant increase in connectivity (measured as increased FA and decreased radial diffusivity) in a network connecting left hemisphere language regions and their right hemisphere analogs (e.g., IFG, caudate nucleus, STG). The most prominent changes occurred in the frontal tracts that cross the anterior-CC, speaking for an increased interhemispheric connectivity in Chinese-learners. The authors also show that FA increases progressively over time and that this increase is related to the level of second language proficiency.

## DISCUSSION

Even if the evidence is still sparse and considerable differences between studies exist, an aggregation of the findings on gray matter changes suggests that structural changes in l-IPC and l-IFG seem to be most consistently related to measures of global second language learning or bilingualism. Both regions have also been repeatedly linked to second language proficiency in studies on functional brain activation (e.g., Chee et al., 2001; Perani et al., 2003; Sakai et al., 2004; Stein et al., 2006, 2009; Raboyeau et al., 2010; Li et al., 2014).

Concerning the learning environment, l-IPC was observed to vary with L2-learning irrespective of the learning setting: The early bilinguals as well as part of the late bilinguals in the Mechelli-study learned L2 through naturalistic exposure while at least another group of the late bilinguals in the Mechelli-study acquired L2 through classroom instruction (personal communication Cathy Price, June 3rd 2014, Andrea Mechelli, June 4th 2014), the latter being also true for participants in the study by Osterhout et al. (2008). Despite this variation, l-IPC-changes have been observed in all three groups, suggesting that these changes seem to accompany L2-acquisition irrespective of learning setting. Even if the l-IPC-changes are more pronounced in early compared to late bilinguals, this variation is most likely attributed to differences in AoA (Mechelli et al., 2004).

Concerning the influence of naturalistic immersion on the l-IFG finding, a group with extensive immersion into an L2-environment (exchange students in Stein et al., 2012) as well as the group with the clearest non-naturalistic, classroom setting (interpreters in Martensson et al., 2012) displayed structural changes in l-IFG. In turn, the early Spanish-Catalan bilinguals in Ressel et al.’s (2012) study did not display l-IFG changes. One might argue that the results of Ressel et al. (2012; where the only difference was observed in bilateral HG) might also be due to the fact that the bilinguals’ two languages mainly differed in phonology, while having a considerable lexical overlap. On the other hand, together with the study by Klein et al. (2014), where the simultaneous bilinguals were the only bilingual group without increased l-IFG thickness, this might also to suggest that it is not immersion as such but rather “age of immersion” that might influence whether structural l-IFG changes occur or not: As the l-IFG is involved in cognitive language control (Abutalebi and Green, 2008; Luk et al., 2011b), controlled retrieval (Rodriguez-Fornells et al., 2009) and morphosyntactic processing (Pliatsikas et al., 2014b), it might be particularly recruited by explicit learning. This learning type, even if directly targeted only by traditional classroom instruction, might also be deployed by late bilinguals during highly immersed learning. Such an interpretation is in line with the observation that younger learners outperform older ones in implicit learning while older learners are more apt to rely on (and better in) explicit learning (DeKeyser and Larson-Hall, 2005; Mu noz, 2006).

Regarding other brain regions reported to vary with global second language proficiency, l-STG and l-MFG were until now only observed to change in a classroom setting (Martensson et al., 2012). The fact that the second study with a classroom setting did not replicate these findings must not be mistaken as conflicting evidence, as Osterhout et al. (2008) only performed a ROI-analysis of l-IPC. When extending the focus to studies on single L2 domains (like, e.g., Li et al., 2014), the only study that observed structural changes in these regions when analyzing L2-learning in a naturalistic setting was conducted by Crinion et al. (2009) and related l-STG changes to the acquisition of a tonal as opposed to non-tonal languages. However, in both regions differential functional activation in L2-processing was also observed in samples with high L2-immersion (e.g., Parker Jones et al., 2012; Archila-Suerte et al., 2013). Furthermore, a study on L2-related WM changes in a highly immersed sample (García-Pentón et al., 2014) reported connectivity changes in a network including STG. In the case of STG, its assumed functional role in phonological processing (Callan et al., 2004; Zheng et al., 2010), additionally undermines the assumption that naturalistic L2 learning should be less effective in inducing STG changes. It thus seems likely that the failure to observe structural changes in these regions in response to naturalistic L2 learning is merely due a lack of research on that issue. Thus, future studies directly comparing naturalistic learning and classroom instruction while controlling for differences in AoA and proficiency level are necessary to determine the influence of L2-immersion on gray matter changes.

Taken together, the studies on WM changes repeatedly reported L2-related changes in the CC, the main anatomical link between left and right hemispheres. Generally, this finding is in line with the observation that the language network in bilinguals seems to be less left lateralized and more bilateral compared to monolinguals (Hull and Vaid, 2006), while the compatibility with the assumption that AoA is the most important factor in determining the degree of lateralization (Hull and Vaid, 2007) seems less clear. Note that most of the data on CC-changes stems from group comparisons between bi- and monolinguals, making it hard to draw inferences about the dynamic of these changes as well as about the role of the precise proficiency level. Regarding the precise portion of the CC that adapts in response to L2-exposure, the anterior and anterior-midbody CC seem to be candidate regions: Coggins reported relative larger anterior-midbody CC, Mohades et al. (2012) observed lower FA-values in the anterior part of CC in bilingual children, and a large part of the regions found to be increasingly interconnected in the study by Schlegel et al. (2012) are anatomically connected via anterior to mid-CC. However, there are still inconsistencies and open questions regarding the factors (e.g., age) influencing structural changes in CC. Furthermore, both, changes in relative volume (Coggins et al., 2004) as well as FA-changes (Mohades et al., 2012) may be due to different axonal characteristics [e.g., myelination, axonal density, axonal caliber, and fiber coherence (Cheng et al., 2010)], thus the precise nature of the underlying adaptation remain to be further explored. Considering the effects of naturalistic exposure, the CC seems to undergo changes in response to naturalistic L2 learning (simultaneous bilinguals in Mohades et al., 2012) as well as during classroom instruction (Coggins et al., 2004; Schlegel et al., 2012).

Another fiber tract adapting when people acquire a second language seems to be the IFOF (Mohades et al., 2012). These results are in line with studies relating the IFOF to semantic processing (e.g., Duffau, 2008; Martino et al., 2010) as well as with studies on WM integrity in elderly bilinguals (e.g., Luk et al., 2011a; but see Gold et al., 2013; for changes in the opposite direction). Very interestingly, when looking closely at the results, IFOF-changes might be most pronounced when L2 is learned through naturalistic exposure: The group with the most pronounced IFOF-effects in the study by Mohades et al. (2012) acquired their L2 through naturalistic exposure and so did the elderly sample in the Luk et al. (2011a) study. Not in line with this interpretation, however, is the absence of IFOF changes in the study by García-Pentón et al. (2014), which examined bilinguals with high levels of L2-immersion. In turn, García-Pentón et al. (2014) reported increased connectivity in a left-sided network comprising frontal regions as well as supramarginal gyrus, thus a network that is partly connected via the superior longitudinal fasciculus (SLF). Consistently, the elderly bilinguals with naturalistic exposure in Luk et al. (2011a) equally displayed SLF-alterations. This might indicate that immersion in L2 (in contrast to pure classroom instruction) has a stronger influence on SLF-changes. A study directly comparing two bilingual groups with different learning experiences however failed to find SLF differences (Mohades et al., 2012). Thus, future studies are needed to enlighten the effects of immersion on WM changes.

## Conflict of Interest Statement

The authors declare that the research was conducted in the absence of any commercial or financial relationships that could be construed as a potential conflict of interest.
